# Oral ketamine may offer a solution to the ketamine conundrum

**DOI:** 10.1007/s00213-023-06480-x

**Published:** 2023-10-26

**Authors:** Megan Dutton, Adem T. Can, Jim Lagopoulos, Daniel F. Hermens

**Affiliations:** Thompson Institute, University of the Sunshine Coast, 12 Innovation Parkway, Birtinya, QLD 4575 Australia

**Keywords:** Oral ketamine, Norketamine, Pharmacokinetics, Bioavailability, Efficacy, Clinical use

## Abstract

Ketamine has received considerable attention for its rapid and robust antidepressant response over the past decade. Current evidence, in clinical populations, predominantly relates to parenterally administered ketamine, which is reported to produce significant undesirable side effects, with additional concerns regarding long-term safety and abuse potential. Attempts to produce a similar drug to ketamine, without the psychotomimetic side effects, have proved elusive. Orally administered ketamine has a different pharmacological profile to parentally administered ketamine, suggesting it may be a viable alternative. Emerging evidence regarding the efficacy and tolerability of oral ketamine suggests that it may be a favourable route of administration, as it appears to obtain similarly beneficial treatment effects, but without the cost and medical resources required in parenteral dosing. The pharmacological effects may be due to the active metabolite norketamine, which has been found to be at substantially higher levels via oral dosing, most likely due to first-pass clearance. Despite bioavailability and peak plasma concentrations both being lower than when administered parenterally, evidence suggests that low-dose oral ketamine is clinically effective in treating pain. This may also be due to the actions of norketamine and therefore, its relevance to the mental health context is explored in this narrative review.

## Introduction

Ketamine, an N-methyl-D-aspartate (NMDA) receptor antagonist, is one of only a handful of drugs that acts primarily on the glutamatergic system. Whilst it has been used as an anaesthetic agent for the past 50 years, only recently has it generated interest in the mental health arena (Nowacka and Borczyk 2019). Its rapid acting antidepressant effects have been well documented in the treatment of depression and other stress related disorders via intravenous (IV) dosing (Murrough et al. 2013; Feder et al. 2014; Taylor et al. 2018). Its rapid effect is related to the pharmacokinetics, with high lipid solubility, low levels of protein binding, fast penetration of the blood–brain barrier, rapid redistribution and rapid elimination (2–3 h post-dosing) (Palucha-Poniewiera 2018). More recently, oral ketamine has garnered attention (Rosenblat et al. 2019) and it potentially provides a more practical and less costly treatment option in a real-world setting, where it is highly scalable to meet clinical demand. Though the bioavailability of oral ketamine is significantly lower at between 17 and 25%, most likely due to first-pass metabolism, compared to intravenous (IV) dosing at 97% (Palucha-Poniewiera 2018) it presents an interesting case showing comparable efficacy to intravenous dosing (Can et al. 2021). Whilst the exact mechanism for this is unknown it has been postulated to be due to the effects of the main metabolite, norketamine. Studies show blood concentrations of norketamine are substantially higher following oral administration than when administered by other routes (Clements and Nimmo 1981, Clements et al. 1982) and this may contribute to the efficacy observed in oral dosing. Additionally, evidence from pain research indicates analgesic effectiveness at lower blood concentrations and a greater longevity of response with oral administration (Grant et al. 1981), than observed in parenteral or intramuscular (IM) dosing, further supporting this notion. Moreover, ketamine’s glutamatergic action is pivotal to both analgesic and antidepressant effects (Peltoniemi et al. 2016), and thus, this review considers the clinical evidence for low-dose oral ketamine, the pharmacological rationale which may underpin the observed treatment effect and the relevance of pain studies for ketamine use in psychiatry. We posit that oral administration of ketamine for depression and suicidality similarly benefits from the norketamine effect observed in analgesia studies and further assert that oral administration of ketamine may be the route of choice, providing desirable clinical effects with less undesirable side effects associated with parenteral dosing. This narrative review was conducted utilising articles found after searches in various databases including SCOPUS, PubMed and Google Scholar with the following key terms: ‘oral’, ‘ketamine’, ‘pharmacokinetics’, ‘pharmacodynamics’, ‘pain’ and/or ‘depression’. Furthermore, manual searches of the reference lists of the papers identified in the database search were undertaken. Finally, the search was conducted in English and only English-language studies were included.

## Clinical use in psychiatry

Reports of ketamine’s antidepressant action date back to the 1970s, with preclinical evidence suggesting that ketamine induced similar antidepressant effects as classic antidepressant drugs, in rodent models. Sofia and Harakal reported that oral ketamine displayed significant antidepressant drug activity in mice over a range of doses up to 40 mg/kg (Sofia and Harakal 1975). Whilst, in 1973, a human study of IV ketamine at 0.2–1 mg/kg was reported to result in an improvement in the condition of 100 psychiatric inpatients (Khorramzadeh and Lofty 1973), but it was not until the year 2000 that Berman et al. conducted a placebo-controlled randomised controlled trial (RCT) and established the antidepressant efficacy of ketamine in patients with depression, reporting a robust and rapid treatment response to a 40-min IV infusion of 0.5 mg/kg ketamine versus saline solution (Berman et al. 2000). Zarate et al. replicated this with a double-blinded, placebo-controlled RCT in 2006 (Zarate et al. 2006). They reported treatment response became evident just 2 h post-infusion and lasted for an average of 7 days, in patients with treatment-resistant depression. Murrough et al. introduced the active placebo midazolam in an RCT, reporting ketamine showed a higher response rate (64%) compared to midazolam (28%) in patients with treatment-resistant depression (Murrough et al. 2013). A meta-analysis of 9 RCTs using IV ketamine further supported ketamine’s clinical efficacy, with a significant improvement in depression scores compared to placebo, in 226 patients with major depressive disorder (MDD) or bipolar depression, with duration of effect in longitudinal studies reported at 2–3 days (Fond et al. 2014). The question of whether repeated dosing could induce a more prolonged effect was considered by Singh et al. (2016). A multicentre double-blind trial assessed the efficacy of twice-weekly or thrice-weekly dosing in 68 patients with treatment-resistant depression, for up to 4 weeks. Both dosing regimens were reported as similarly effective, whilst the clinical effect persisted for up to 15 days (Singh et al. 2016). Subsequent studies have shown significant anti-suicidal effects, with reductions in suicidal ideation (Grunebaum et al. 2017, 2018) and anhedonia reported (Murrough et al. 2015; Ballard et al. 2017).

In comparison, reports of oral ketamine have been slower to emerge, potentially due to concerns regarding lower bioavailability (compared to IV), which has been assumed to impact treatment response. However, although numbers are limited, reports of oral ketamine for mental health treatment use, have been largely positive. In 2010, a retrospective study reported oral ketamine (0.5 mg/kg) use in 14 palliative care patients who had comorbid depression and anxiety symptoms. All 8 who completed the 28-day trial reported significant improvement in their mood and anxiety symptoms, with no serious adverse events reported (Irwin et al. 2013). A further early report of 2 patients with chronic suicidal ideation and at least 2 significant past suicide attempts, who received oral ketamine (0.5–3 mg/kg) as an augmentation to existing treatment, indicated an initial improvement within 24 h of the first treatment (De Gioannis and De Leo 2014). Furthermore, the clinical improvement was reported to be sustained with remission of symptoms after additional treatments (every 2–4 weeks) and a high degree of tolerability and no adverse events. A 2019 systematic review on the efficacy and tolerability of oral ketamine found only 13 published articles including 2 RCTs (Rosenblat et al. 2019). They reported the studies were largely low quality, with a high risk for bias and noted heterogeneity in the treatment regimens further hindered analysis. However, the limited evidence indicated that oral ketamine has significant antidepressant effects and is well tolerated, though onset of treatment effect may not be as rapid as observed with IV ketamine (Rosenblat et al. 2019). Whilst, a recent (2023) systematic review of twenty-two studies including four RCTs, a case review, five open-label trials and six retrospective chart review studies, which involved 2336 patients with depression, concluded that significant clinical benefit was derived from oral ketamine treatment and that treatment was well tolerated, with no serious adverse effects reported (Meshkat et al. 2023). Additionally, our group has recently reported a study in which weekly subanaesthetic oral ketamine resulted in similar positive outcomes in a highly complex clinical population (*n* = 32), with chronic suicidality (Can et al. 2021). Tolerability and feasibility were considered as key aspects of the oral dosing and significant neurobiological outcomes which correlated with the clinical results, were subsequently reported across the six-week treatment period (Gallay et al. 2021; Can et al. 2023a, b). Moreover, anecdotally, the family of participants reported life-changing benefits, which in some cases were sustained well beyond the trial period. Thus, though studies on oral ketamine are limited in numbers and with significant design limitations, early outcomes in human subjects are promising. Additionally, preclinical and clinical evidence strongly suggests that the primary metabolites contribute to ketamine’s therapeutic effects, with norketamine significantly contributing to ketamine’s actions, particularly when administered orally (Grant et al. 1981; Ebert et al. 1997; Shimoyama et al. 1999).

## Safety

Whilst it has been shown that oral ketamine is relatively safe in research settings, it is still to be established in a real-world community setting. Though generally considered to be well tolerated, concerns regarding psychotomimetic side effects and abuse potential have been reported (Krystal et al. 1994; Zarate 2022) and non-medical ketamine use (abuse) has been associated with significant urological complications such as dysuria, increased frequency and urgency, incontinence, haematuria and ketamine-associated ulcerative cystitis, which can be irreversible (Ng et al. 2021). Ketamine has been shown to be toxic to the urothelial cells in the bladder and evidence from cystoscopy revealed oedema and epithelial inflammation in populations using long-term non-medical ketamine (Chu et al. 2008). Imaging of non-medically prescribed users further show bladder wall thickening and perivesicular inflammation with one report indicating a case of urinary urgency and incontinence at subanesthetic dosing levels (0.1 mg/kg per h IV over 12 h) (Mason et al. 2010; Vickers et al. 2017). Though evidence suggests most non-medical use of ketamine is via nasal insufflation (30–400 mg), there are reports of non-medical oral ketamine (100–500 mg) use, with plasma concentrations of 50–200 ng/ml enhancing sensory perception, emotional connectedness, feelings of unreality and out of body experiences, in a dose-dependent manner (Zanos et al. 2018). At peak plasma levels, ketamine may induce extreme dissociative experiences characterised by altered states of consciousness and sensory detachment, with these effects often accompanied by blurred vision, dizziness, vomiting and nausea, palpitations and chest pain (Wolff and Winstock 2006). However, the majority of ketamine’s side effects are dose dependent and demonstrated to be transient and self-resolving (Loo et al. 2016) and despite some reinforcing effects, ketamine dependence is considered to be a rare occurrence (Blier et al. 2012). Furthermore, whilst no studies have directly compared ketamine abuse liability across different routes of administration, oral dosing is considered to be less attractive in this regard, due to slower onset of effect when compared to IV and nasal insufflation (Muetzelfeldt et al. 2008). Indeed, our group has identified a pattern of sensitivity where human participants in a longitudinal study required a lowering of dose and this was particularly evident in those who were responsive to the rapid-acting effects (Can et al. 2023a, b). However, caution should be exercised in treating patients with ketamine, particularly in contexts outside of research and where concomitant opioid medications or alcohol use is present. In these situations, a high degree of monitoring for dependence is warranted (Freedman et al. 2018).

Concerns regarding neurotoxic effects have emerged in both preclinical and clinical studies in substance use disorder models/populations, whereby daily high-dose ketamine use has been associated with specific neuropathologies, including Olney lesions (Nogo et al. 2022). Long-term repeated treatment or single high doses of ketamine in rodents also indicate the potential for selective irreversible damage, though these changes were not apparent at lower, clinically relevant doses (Zanos et al. 2018). Additionally, studies in non-human primates indicate a pattern of reduced white matter integrity in fronto-thalamo-temporal connections after 3 months of daily IV administration (Li et al. 2017). Taken together preclinical and clinical evidence indicates specific neuropathologies may be associated with chronic high-dose ketamine exposure, exceeding therapeutic subanaesthetic dosing and given the increasing interest in ketamine use, these concerns require further investigation.

Evidence regarding ketamine’s safety and utility in a community setting has been reported in the extant pain literature. Ketamine has been used as a take-home self-administered treatment in patients with chronic pain, with efficacy and safety data reported. A 5-year retrospective study reported on 51 patients with intractable pain, who were transitioned from IV ketamine infusion to self-administered oral treatment in the home (Marchetti et al. 2015). Daily treatment began at 0.5 mg/kg and titrated up, with 2 mg/kg reported as the mean effective oral dose, with treatment continued for 3 months, followed by a progressive withdrawal. Half of the patients reported adverse effects and 8 ceased treatment due to tolerability issues with three withdrawals due to headaches and four due to psychotomimetic side effects (Marchetti et al. 2015). In a double-blind, RCT of 40 patients with mild to moderate depression and chronic pain, oral ketamine (150 mg per day in 3 divided 50 mg doses) was compared to the same dose of diclofenac across a 6-week treatment period (Jafarinia et al. 2016). Medication was self-administered and adherence was monitored through patient self-reports and weekly capsule counts. Safety data showed that oral ketamine was safe and well tolerated, with no difference in adverse effects between the two groups reported and no patient withdrawal during the treatment period. Both treatments produced similar analgesic efficacy, but ketamine showed a significantly greater improvement in depression (as measured using the Hospital and Anxiety Depression Scale) scores post-treatment (Jafarinia et al. 2016).

However, despite increasing numbers of positive reports, concerns have persisted regarding ketamine’s side effects (Zarate 2022), and thus, efforts have been made to identify alternate targets for drug discovery, based on ketamine’s mechanism of action, with investigations undertaken into candidate drugs which capture ketamine’s beneficial effects, without its less desirable features. NMDA receptor antagonists were thoroughly investigated and whilst some demonstrated partial effectiveness, they lacked ketamine’s rapid and robust effect, as well as its capacity to illicit a sustained response after a single dose and its broad therapeutic potential to treat other stress-induced disorders (Zarate and Machado-Vieira 2017). Furthermore, investigations into AMPA-positive modulators showed variable results with toxicity concerns (Menniti et al. 2013) whilst studies into other glutamate receptor modulating drugs such as glycine site modulators, sub-unit specific NMDA receptor antagonists and mammalian target of rapamycin complex 1 (mTORC1) activators have yielded modest results, when compared to racemic ketamine (Henter et al. 2021).

## Chemical properties and pharmacokinetic profile

Chemically, ketamine is a 2-(2-chlorophenyl)-2-(methylamino)-cyclohexane and phencyclidine derivative, with a molecular weight of 237.73 (Zanos et al. 2018). It is highly lipid soluble, but also soluble in water to 20% and has a chiral structure with equal amounts of the two optical enantiomers, S( +) -ketamine and R( −) -ketamine, due to an asymmetrical carbon atom in the C2 position (Schmid et al. 1999; Peltoniemi et al. 2016). Racemic ketamine, as a HCL salt, is commonly utilised for use in humans, though both the S ( +) and R ( −) enantiomers have been investigated, in an effort to manage the side effect profile of parenterally administered ketamine (Zanos et al. 2018; Kaur et al. 2021). Recent evidence indicated R ( −) ketamine may exert neuroprotective effects and longer lasting antidepressant effects, when compared to S ( +) ketamine (Passie et al. 2021).

Analyses report the bioavailability of oral ketamine to be between 16 and 29%, most likely due to extensive first-pass metabolism by the liver, with peak plasma concentrations occurring within 20–120 min (Grant et al. 1981; Clements et al. 1982). After systemic absorption, ketamine’s high degree of lipid solubility and low levels of plasma protein binding (10–30%) facilitate rapid transfer across the blood–brain barrier and distribution into highly perfused tissue, including the brain. A large steady-state volume of distribution (160–550 L/70 kg) follows (Peltoniemi et al. 2016), with the initial effects subsiding as the drug moves from highly perfused tissue into less perfused compartments. Biotransformation occurs in the liver via the cytochrome p450 liver enzyme system, with the major metabolic pathway nitrogen demethylation of the parent drug, by hepatic enzymes CYP2B6 and CYP3A4, to norketamine (Mion and Villevieille 2013). Demethylation occurs in a stereoselective manner with S- ketamine selectively metabolised by CYP3A4 more rapidly than R ketamine (Zanos et al. 2018). Norketamine is subsequently hydroxylated to 6-hydroxynorketamine by CYP2B6 and CYP2A6 enzymes or to dehydroxynorketamine (DHNK) by CYP2B6 (Mion and Villevieille 2013). Elimination occurs through the kidneys after norketamine and other metabolites undergo glucuronide conjugation into water soluble compounds and are finally excreted in the urine and bile, with only a minor percentage of unchanged drug (Haas and Harper 1992). A secondary metabolic pathway is the direct hydroxylation of ketamine to hydroxynorketamine (HNK) and in animal models this may additionally occur in the intestines, liver and lungs (Zanos et al. 2018). Notably, oral administration is associated with higher serum levels of the active metabolite norketamine, compared to other routes of administration. Thus, the bioavailability of ketamine 0.5/mg/kg given orally, is reported at approximately 20% of the availability of parenterally administered treatment, such as an IV infusion. However, importantly, Chong et al. noted that the oral bioavailability of ketamine may reach 59 ± 16%, when allowance is made for norketamine’s contribution (Chong et al. 2006). Additionally, Grant et al. (1981) noted that though the bioavailability of norketamine is similar between the intramuscular and oral routes of administration, significantly higher peak plasma concentrations are found after oral administration (see Table 1).

**Table 1:** Pharmacokinetic parameters for racemic ketamine and norketamine after different dosing routes

Author	Route	Dose	Parameter	Ketamine	Norketamine	Comments
Chong et al. (2006)^a^	IV	10 mg	C_max_ (ug/ml)	156 ± 161	38 ± 29	*N* = 6 neuropathic pain
			AUC (ug hr/ml)^b^	13.4 ± 2.4	13.0 ± 6.9	
			T_max_ (min)	14.4 ± 17.4	36 ± 42	
	SL	25 mg	C_max_ (ug/ml)	28.6 ± 6.6	66 ± 29	
			AUC (ug hr/ml)^b^	4.0 ± 1.9	9.9 ± 5.3	
			T_max_ (min)	45.6 ± 30.6	114 ± 60.0	
	Oral	25 mg	C_max_ (ug/ml)	22.8 ± 12.8	82 ± 34	
			AUC (ug hr/ml)^b^	3.1 ± 0.7	12.1 ± 5.3	
			T_max_ (min)	57.6 ± 48	96 ± 54	
Grant et al. (1981)	IM	0.5 mg/kg	C_max_ (ng/ml)	240 ± 50	90 ± 10	N = 6 healthy volunteers
			AUC (ng hr/ml)	-	31.3	
			T_max_ (min)	22	78	
	Oral	0.5 mg/kg	C_max_ (ng/ml)	45 ± 10	200 ± 44	
			AUC (ng hr/ml)	4.8	36.7	
			T_max_ (min)	30 ± 5	60 ± 13	
Yanagihara et al. (2003)^a^	IV	20 mg	C_max_ (ng/ml)	-	38.1 ± 6.1^e^ 34.9 ± 6.4^f^	*N* = 3 healthy volunteers
			AUC (ng hr/ml)	138 ± 15.6^c^126.3 ± 15.1^d^	173.8 ± 26.5^e^149.8 ± 9.5^f^	
			T_max_ (min)		23.3 ± 5.8^e^23.3 ± 5.8^f^	
	Oral	50 mg	C_max_ (ng/ml)	42.6 ± 13.3^c^40.4 ± 14.2^d^	188.1 ± 4.7^e^ 172 ± 13.7^f^	
			AUC (ng hr/ml)	72.3 ± 22.4^c^64.9 ± 27.4^d^	533.8 ± 47.1^e^494.9 ± 73^f^	
			T_max_ (min)	33.3 ± 15.3^c^30 ± 30^d^	50 ± 17.3^e^50 ± 17.3^f^	
	SL	50 mg	C_max_ (ng/ml)	61.6 ± 21.3^c^ 56.9 ± 22.8^d^	123.4 ± 10.7^e^109.7 ± 16.6^f^	
			AUC (ng hr/ml)	108.8 ± 17.3^c^110.5 ± 18.4^d^	462.9 ± 65.2^e^408.7 ± 35.1^f^	
			T_max_ (min)	40.0 ± 20.0^c^36.7 ± 20.8^d^	113.3 ± 70.2^e^93.3 ± 75.7^f^	
	IN	25 mg	C_max_ (ng/ml)	29.4 ± 16.5^c^29.3 ± 13.8^d^	33.1 ± 25.3^e^29.4 ± 21.3^f^	
			AUC (ng hr/ml)	76.8 ± 27.9^c^72.7 ± 17.5^d^	115.9 ± 17.6^e^100.7 ± 39.6^f^	
			T_max_ (min)	22.5 ± 9.6^c^17.5 ± 5.0^d^	120.0 ± 52^e^100 ± 17.3^f^	

^a^Each value represents the mean ± standard deviation

^b^Area under the plasma concentration–time curve from baseline to 8 h

^c^(R)-ketamine

^d^(S)-ketamine

^e^(R)-norketamine

^f^(S)-norketamine

AUC area under the plasma concentration–time curve; C_max_ maximum plasma concentration; t_max_ time to reach C_max_; IV intravenous, SL sublingual; IM intramuscular; IN intranasal.

Similar findings were reported in a small comparison study of ketamine and norketamine plasma concentrations after IV, oral, intranasal, sublingual and rectal administration in healthy volunteers. The area under the plasma concentration–time curve of norketamine was highest for oral administration (500 ng h/ml) in both enantiomers, followed by sublingual and rectal administration (280–460 ng h/ml), with intranasal administration delivering low concentrations of norketamine (100 ng h/ml) in both enantiomers (Yanagihara et al. 2003). Furthermore, the pain literature indicates that the analgesic effects of IM and IV ketamine are observed to occur at plasma levels of 100–200 ng/ml, whereas effective analgesia in oral dosing is achieved at just 40 ng/ml (Grant et al. 1981; Blonk et al. 2010). Taken together, this suggests norketamine may play a significant role in the analgesic effect of orally administered ketamine and this could reasonably also be the case in the antidepressant effect of oral ketamine. Moreover, evidence indicates the high levels of norketamine may contribute to the extended duration of treatment effect after oral administration in pain studies, due to norketamine’s slower rate of elimination, and this is further coupled with enhanced treatment tolerability due to norketamine’s favourable safety profile (White et al. 1982, Holtman et al. 2008.

Ketamine has a high rate of clearance and a short elimination half-life of 2–4 h; however, the therapeutic effect may be prolonged through the active metabolites DHNK, HNK and norketamine, which is reported to have a half-life of up to 12 h (Soto et al. 2012; Zanos et al. 2018). Though recent evidence has questioned the antidepressant efficacy of secondary HNK metabolites (Abdallah 2020). A study on bipolar depression reported evidence of metabolites DHNK and HNK, 3 days after administration, whilst a paediatric study found norketamine was present in the urine 14 days after administration and ketamine 11 days post-administration (Adamowicz and Kala 2005). Furthermore, when compared to adults, children have been shown to metabolise ketamine twice as rapidly (Haas and Harper 1992), suggesting a prolonged exposure to norketamine after oral administration in adults, which may contribute to the therapeutic effect. Additionally, repeated dosing may result in a reduced elimination, with one study of three 0.75–1.59 mg/kg single infusions across a 2-year period reporting decreased clearance on subsequent treatments compared to the first treatment, despite the third treatment being 2 years later (Adamowicz and Kala 2005). Moreover, norketamine accumulation may account for the majority of ketamine’s effect in repeat dosing, and this may explain the reduced need for ketamine in clinical settings, over time (Mion and Villevieille 2013).

However, oral ketamine is more susceptible to individual factors which may affect the bioavailability, including the drug preparation, cytochrome enzyme phenotypes and liver enzyme induction. Ketamine metabolism is a function of liver blood flow with a high hepatic extraction ratio (0.9), and thus, its clearance is sensitive to changes in hepatic blood flow (Haas and Harper 1992). Further factors affecting clearance include the high degree of variability in the expression and catalytic action of the CYP enzymes responsible for ketamine’s metabolism in humans (Nottage et al. 2023). Genetic variability, including CYP2B6 genotypes, may also contribute to the heterogeneity of treatment response, particularly in regard to side effects. The CYP2B6*6 allele is shown to significantly reduce ketamine metabolism resulting in greater plasma concentrations and an associated increase in adverse effects (Li et al. 2015). Furthermore, the influence of enzyme inhibitors and inducers need to be considered, with both shown to have a significant effect on ketamine concentrations after oral administration in healthy volunteers (Peltoniemi et al. 2012). Enzyme induction by rifampicin was shown to significantly reduce the plasma concentrations of both S-ketamine and S-norketamine after oral administration, with the mean area under the plasma concentration–time curve (AUC) decreased by 86% and the peak plasma concentration by 81% (Peltoniemi et al. 2012). Thus, concomitant use of drugs which interfere with ketamine’s CYP3A4- and CYP2B6-mediated elimination will affect the bioavailability of oral treatment.

Additionally, the form of administration may contribute to the treatment outcomes due to differing compounding formulations, as oral ketamine is not commercially available. Oral administration, utilising a sublingual ketamine wafer preparation, has been shown to provide an absolute bioavailability of 29%, notwithstanding the norketamine component, in a study of eight healthy male volunteers (Rolan et al. 2014). Peak plasma concentration was reported at 45 min, though ranged from 25 min to 1 h. Interestingly, the first quantifiable plasma ketamine concentration was recorded within just 5 min when taken orally, which is comparable to IV dosing. However, protocols for oral dosing require further investigation with studies demonstrating considerable heterogeneity in dosing regimens to date (Table 2). Given the pharmacokinetic profile of orally administered racemic ketamine, daily dosing may not be required. Our work (Can et al. 2021) corroborates earlier studies and shows promising evidence for single weekly dosing (De Gioannis and De Leo 2014), though this requires larger scale replication.

**Table 2:** Dosing regimens in orally administered ketamine clinical trials

Author	Study design	Sample size	Dose	Dosing interval	Indication	Outcomes
Enarson et al. (1999)	Open-label retrospective review	*n* = 21	Commenced at 50 mg bd titrated up by 40 mg per day until efficacy achieved or tolerability issues emerged. Up to 500 mg in 1 patient. Average dose 220 mg per day for over 1 year.	Daily	Chronic neuropathic pain	Reduction in pain. Decreased use of other analgesia in 15% of cohort. Nearly half (42%) ceased treatment due to intolerable side effects.
Lara et al. (2013)	Open-label case series	*n* = 26	10 mg sublingual	20 doses every 2, 3 or 7 days	Treatment-resistant unipolar or bipolar depression	75% showed significant response with improved mood, cognition and sleep. No dissociative, manic or psychotic symptoms reported. Lightheadedness reported in first 30 min. 1 patient ceased treatment after 1 dose due to no response.
Marchetti et al. (2015)	Open-label 5-year retrospective study	*n* = 55	0.5 mg/kg initially, titrated up in 15–20-mg increments with mean effective dose 2 mg/kg	Daily, tds or qid for up to 3 months	Intractable pain-neuropathic pain 60%, community management	Mean effective dose 2 mg/kg. Reduction or cessation of pain in 66% of patients. 49% experienced no adverse effects, 8 (15%) patients ceased treatment due to side effects.14% had malaise, dizziness, hallucinations, 13% reported headache, 11% sedation.
Al Shirawi et al. (2017)	Open-label case series	*n* = 22	50 mg titrated up by 25 mg every 3 days according to response and tolerability	Every 3 days for minimum 4-week period	Treatment-resistant depression	Shown to be safe and well tolerated but limited efficacy with 30% responder rate. Most frequent adverse event was acute dissociation (41%), also dizziness (23%), blurred vision (18%), numbness (14%), sedation (4%), nausea (4%), insomnia (4%).
Irwin et al. (2013)	Open-label proof-of-concept	*n* = 14	0.5 mg/kg	Daily for 28 days	End of life, hospice care	Significant improvement in both depression (*p* = 0.002) and anxiety (*p* < 0.001). 12.5% reported diarrhoea, trouble sleeping, trouble sitting still. Somatic symptom severity decreased significantly from baseline-neurological, *p* = 0.022, psychiatric/behavioural *p* = 0.002, gastrointestinal *p* = 0.044.
Hartberg et al. (2018)	Open-label retrospective study	*n* = 37	0.5–7 mg/kg	2 doses initially, 3 h apart, then twice a week. Titrated up by 20–50% per treatment.	TRD and PTSD	70% reduction in inpatient days and 65% reduction in admissions. Stable dose over time, no evidence of tolerance and no significant side effects.
Buvanendran et al. (2018)	Open-label pilot trial	*n* = 3	1 mg/kg reduced to 0.5 mg/kg on the second day of treatment	Three times a day for 3 days perioperatively	Acute pain management post amputation	Well tolerated with no adverse effects. Opioid sparing.
De Gioannis and De Leo 2014)	Open-label case reports	*n* = 2	0.5–6 mg/kg, two case reports treated at 3 mg/kg	Twice a week for up to 12 months	TRD, suicidality	Remission of suicidality. Well tolerated with no adverse events.
Cvrcek (2008)	Open-label trail	*n* = 32	30 mg	Five times a day for 3 months	Pain, postherpetic neuralgia, diabetic polyneuropathy	Significant side effects, 12.5% (4/32) ceased treatment due to side effects. 25% drowsiness, 19% dry mouth, also dizziness, sedation, loss of appetite, nausea, vomiting
Can et al. (2021)	Open-label pilot trial	*n* = 32	0.5–3 mg/kg flexible dosing, titrated up or down by 0.2–0.7 mg/kg to maximum 3 mg/kg across 6 weeks of treatment	Weekly dosing for 6 consecutive weeks	Chronic suicidality	Significant reduction of chronic suicidality (69%) after 6 weeks of treatment (*p* < 0.001). Well tolerated with no severe adverse events. 22% reported decreased energy and fatigue post-treatment followed by anxiety, poor concentration, restlessness, general malaise, dry mouth, dizziness and tremors, and 94% rated their symptoms to be mild.
Domany et al. (2019)	Randomised,double-blind, placebo-controlled trial	*n* = 22 ketamine *n* = 19 control	1 mg/kg ketamine	Three times a week for 21 days	Treatment-resistant depression	Well tolerated. Rapid and persistent reduction in depressive symptoms (*p* < 0.005).
Glue et al. (2020)	Open-label flexible dosage uncontrolled trial	*n* = 7	60–240 mg, in 60 mg extended release tablet Day 1: 60 mg at 8 am, 60–120 mg at 8 pm depending on response, safety and tolerability. Day 2–4: 60–240 mg 12 hourly.	12 hourly, for 72 h	Treatment-resistant depression/anxiety	All patients (*n* = 7) (100%) had > 50% mood improvement. Safe and well tolerated.
Arabzadeh et al. (2018)	Randomised, double-blind placebo-controlled trial	*n* = 45 ketamine *n* = 45 placebo	50 mg in divided dose of 25 mg	Daily	MDD	Adjunctive treatment with sertraline or sertraline and placebo. Ketamine group significantly greater early improvement (85%) compared to placebo (42%). Well-tolerated, side effects no difference between groups.
Jafarinia et al. (2016)	Randomised, double-blind, controlled, parallel group trial	*n* = 20 ketamine *n* = 20 diclofenac	Fixed dose 150 mg, in three 50 mg doses	Daily for 6 weeks	MDD in chronic pain patients	Significantly lower HDRS (*p* = 0.005) and HADS (*p* = 0.005, week 3, *p* = 0.003, week 6) No serious adverse events, no discontinuation due to drug effects, main side effects blurred vision 5% (*p* = 1.0) tremor 5% (*p* = 1.0). Greater efficacy reported at 6 weeks than 3 weeks, endorsing potential long-term treatment option.

Thus, oral ketamine is highly susceptible to pharmacokinetic interactions. Gene-drug interactions and differential drug metabolism due to cytochrome enzyme polymorphisms may affect the efficacy and safety and further impact dosing requirements in clinical practice (Zanos et al. 2018).

## Pharmacodynamic effects

Pharmacokinetic studies have reported delayed time to maximum plasma concentrations in oral ketamine dosing compared to other routes; however, the therapeutic effect may be more sustained with oral dosing, in line with the pharmacokinetics of the oral compound (Grant et al. 1981; Chong et al. 2006). Orally administered ketamine undergoes significant first-pass clearance, resulting in lower circulation concentrations and delayed onset of therapeutic effects. However, norketamine has a slower clearance rate and, thus, is thought to be responsible for the more persistent therapeutic effects observed after oral dosing (Mion and Villevieille 2013). Ketamine pharmacodynamics, more generally, have been poorly studied in a mental health context, with limited data regarding optimal AUC values which correlate with the therapeutic effects, pharmacodynamic target engagement and clinical efficacy and toxicity. Additionally, neuronal mechanisms are still to be fully characterised (Lazarevic et al. 2021). Here, we summarise relevant evidence regarding key molecular targets and mechanisms associated with ketamine’s therapeutic effects (see Zanos et al., for a detailed review (Zanos et al. 2018)).

The neuropharmacology of ketamine is complex as it targets several receptors in humans, including nicotinic, muscarinic, mu, delta and kappa opioid receptors, as well as interacting with monoaminergic and voltage gated calcium channels (Mion and Villevieille 2013). It also blocks peripheral and central nervous system sodium channels and further acts as a non-competitive antagonist at the phencyclidine binding site in the NMDA receptor complex channel, where it induces a use-dependent blockade, paradoxically disinhibiting glutamate release (Abdallah et al. 2018). Ketamine is shown to increase the cortical rate of conversion of ^13^C-glutamate to ^13^C-glutamine, which is indicative of glutamate release (Abdallah et al. 2018) and through its action at GluN2B subunits enhances synaptic connectivity and plasticity, through increased levels of brain derived neurotrophic factor and increased shuttling of AMPA receptors to the synapse (Krystal et al. 2019). Enhanced activation of the AMPA receptor is considered critical to ketamine’s action and this is thought to work synergistically with the NMDA receptor at the tripartite glutamatergic synapse (Zarate and Machado-Vieira 2017). Thus, ketamine’s initial NMDA receptor blockade increases glutamate release from synapses and the subsequent preferential activation of AMPA facilitates the plasticity signalling cascades, particularly in the cortico-limbic-striatal circuitry, where it enhances synaptic strength (Abdallah et al. 2018). The resulting downstream plasticity effects are responsible for ketamine’s longer lasting effects (beyond actual drug levels) such as altered gene expression and protein regulation and these are reviewed by others (Zarate and Machado-Vieira 2017; Dutton et al. 2022).

Thus, the role of the NMDA receptor in nociception, as well as the neural plasticity processes implicated in mental disorders, has prompted increasing interest in understanding ketamine’s mechanisms of action. Its unique non-competitive antagonism at the NMDA receptor is use dependent, with agonism at a separate receptor site facilitating binding (Mion and Villevieille 2013). Also, the open state is required for ketamine to subsequently dissociate from the binding site, with entrapment in the receptor channel a potential mechanism for ketamine accumulation (Schmid et al. 1999; Mion and Villevieille 2013). Hence, ketamine may be considered a high entrapment antagonist (86%) with a slow off-rate, creating a prolonged tonic blockade and resulting alterations to physiological functions and subsequent clinical effects (Sleigh et al. 2014). Additionally, NMDA receptor activation plays a fundamental role in long-term potentiation but also in opioid hyperalgesia and its analgesic effects are also thought to result from the non-competitive antagonism of the ionotropic NMDA receptors, which are involved in enhanced nociceptive processing in the spine and supraspinal regions (Haas and Harper 1992). NMDA receptor blockage effectively reduces signal propagation in pain neural circuitry resulting in effective analgesia. Opioid receptor binding additionally mediates the analgesic response, with ketamine preferentially binding to mu receptors, and this is thought to be at least partially associated with the antidepressant effect, though potentially through modulation of endogenous opioid pathways with lower risk of dependence (Grunebaum et al. 2020). Though some suggest ketamine’s effects are not analgesic in the traditional sense of the word, rather the NMDA receptor effects reduce ‘wind up’ and central sensitisation, resulting in an additive effect in combination pain management (Schmid et al. 1999). Whilst its distinctive dissociative anaesthetic effects, characterised by profound amnesia, catalepsy and analgesia, are attributed to a functional and electrophysiological separation between the limbic and thalamoneocortical systems (Haas and Harper 1992; Peltoniemi et al. 2016). The dissociative state arises from the brains inability to differentiate afferent impulses because of altered sensory cortex input, with patients in a non-communicative cataleptic state (Marchetti et al. 2015). Imaging studies have further indicated that physical pain activates similar brain areas as emotional pain (i.e. depression, suicidality), with both activating the insula and somatosensory cortex, and the antidepressant effect is thought to contribute to the analgesic response (Romero-Sandoval 2011; Mion and Villevieille 2013). Ketamine further enhances the inhibitory descending serotonergic pathway (Mion and Villevieille 2013). Recent evidence suggests a role for the endocannabinoid system, with CB1 receptor activation implicated in the analgesic effect of ketamine (d’Andrea et al. 2023), whilst blockade of lateral habernula NMDA receptor-dependent burst firing induced significant antidepressant effects and this may be due to ketamine’s modulation of hippocampal theta-band frequencies with resulting disinhibition of downstream monoaminergic reward centres (Yang et al. 2018). Ketamine has been further shown to produce significant increases in neural gamma oscillations in EEG studies and this potential pharmacodynamic biomarker is associated with clinical antidepressant effects (Gilbert and Zarate 2020). Interestingly, a clinical study reported low-range gamma oscillations were associated with higher levels of HNK metabolites and these were associated with poorer clinical outcomes (Farmer et al. 2020) whilst norketamine exposure positively correlated with improved clinical outcomes in another recent EEG study and oral administration of the similar acting glutamatergic antidepressant d-cycloserine (DCS) produced more sustained frontal enhancements compared to the transient effects of IV ketamine, in line with the pharmacokinetics of the oral compound (Nottage et al. 2023).

## Discussion and future directions

The emergence of ketamine as a rapid-acting antidepressant has perhaps been the most significant event in the treatment of depression and suicidality in the last decade. However, whilst numerous studies report ketamine’s robust rapid-acting effects, the optimal route of administration is still being elucidated. Recently, oral ketamine has garnered attention for its ease of administration, excellent tolerability and comparable efficacy (Can et al. 2021). Intravenous infusions show efficacy but involve medical complexity (Bahji et al. 2021) and are costly in comparison to orally administered treatment. Additionally, the ambulatory outpatient treatment associated with oral administration offers a more practical and accessible solution that does not require hospital resources. Furthermore, a recent systematic review examined the influence of formulation and route of administration on ketamine’s safety and tolerability, reporting the extent of first-pass metabolism and time to peak plasma concentrations were significantly associated with cardiovascular changes and psychotomimetic effects and concluding that formulations which maximise first-pass metabolism and delay absorption (such as oral formulations) may optimise safety and tolerability (Glue et al. 2021), whilst maintaining the desired clinical effects. Whilst evidence from a recent systematic review on the efficacy of oral ketamine in *n* = 1667 MDD patients indicated significant clinical improvement without serious adverse events, though limitations included a small number of RCTs and methodological bias (Meshkat et al. 2023). Moreover, two clinical studies reported increased hydroxynorketamine exposure after ketamine infusion was associated with poorer clinical response both short and long term in treatment-resistant depression (Farmer et al. 2020) and suicidal depression (Grunebaum et al. 2019), whilst a high-frequency EEG study reported positive correlations between maximum norketamine concentrations and antidepressant potential (Nottage et al. 2023). However, scepticism has remained regarding the bioavailability of oral ketamine which is perceived as an impediment to treatment. Though evidence is limited, the pharmacokinetic/pharmacodynamic profile of oral ketamine is shown to support oral dosing as a viable treatment option with high levels of metabolism and the more persistent action of the active metabolite norketamine, producing promising clinical outcomes with fewer adverse effects (Glue et al. 2021; Meshkat et al. 2023).

Additionally, ongoing concerns regarding ketamine’s abuse potential may be overstated, with little data presented in support of dependency liability and furthermore, evidence suggests interactions at opioid receptors are ‘opioid sparing’, potentially due to ketamine’s NMDA receptor effects, which may protect against tolerance (Swainson et al. 2022). However, contrarily, Williams et al. (2018) report that opioid pathways are integral to ketamine’s action, stating that 50 mg of naltrexone significantly blocked ketamine’s antidepressant effects, though others have suggested this work should be treated with caution, after a small study showed naltrexone 380 mg failed to attenuate ketamine’s antidepressant effects (Yoon et al. 2019) and earlier preclinical evidence suggests that ketamine isomers may be weak partial agonists at the mu opioid receptor (Hirota et al. 1999). Thus, the role of opiodergic and other mechanisms in the therapeutic and safety profile of ketamine is still to be fully investigated via sufficiently powered studies. Taken together, with demonstrated tolerability and safety, and comparable efficacy, low-dose oral ketamine shows some advantages over other routes, and therefore, any concerns about its clinical use should be reconsidered. However, judicious clinical prescribing is warranted, as with other well-established treatments such as stimulants or benzodiazepines with potential for misuse, risks and benefits should be considered together (Swainson et al. 2022).

Thus, the evidence presented here from psychiatric and pain research suggests that the pharmacological nature of orally administered racemic ketamine and the resulting high levels of norketamine may account for its reported therapeutic effects. That is, despite reports of low bioavailability and peak plasma concentrations, there is increasing evidence in clinical populations showing the considerable promise of oral ketamine. Indeed, though it is yet to be thoroughly and rigorously examined, early evidence suggests oral ketamine is worthy of larger scale investigations for mental health treatment uses, due to both improved tolerability and possible sustained treatment effects associated with the pharmacokinetics of the oral formulation. Our group reported 50% of trial participants with MDD and suicidality demonstrated a prolonged treatment response, 4 weeks after the final oral ketamine dose; however, this requires replication (Can et al. 2021). Thus, future studies are particularly required in long-term use, where oral ketamine may offer greater utility, to establish treatment protocols and examine the potential for adverse effects including neurotoxic effects, with some reports indicating the potential for an associated decline in cognition and memory (Liu et al. 2019; Driver et al. 2022) or addiction (Schak et al. 2016) in longer term use.

Based on the evidence to date, there is more work to be done regarding dosing and formulations. Further investigations into the optimal compounding formulas for oral preparations and treatment regimens are also of primary importance. Whilst the oral preparations are demonstrated to be efficacious with excellent general tolerability due to reduced psychotomimetic effects, the issue remains that absorption and bioavailability are highly variable. It could be argued that the extensive metabolism to norketamine is a desirable feature of oral administration, resulting in a therapeutic effect which is slower to develop but more sustained, due to the suspected, though yet to be fully investigated, activity of norketamine. Additionally, absorption may be improved by the addition of mucosal adhering agents to oral formulations. This addition may facilitate increased absorption, thus potentially contributing to a reduction in individual pharmacokinetic variability. Mucoadhesion is shown to improve absorption through prolongation of residence time in the gastrointestinal tract, resulting in improved bioavailability (Shaikh et al. 2011). This feature is of particular benefit to drug molecules that are not suited to oral dosing, including those that undergo extensive first-pass metabolism. Our group have utilised a mucosal adhering agent in the compounded oral solution, potentially contributing to treatment outcomes and this is an area which requires further investigation. Interestingly, a study on sublingual dosing observed a significant reduction (35–50%) in the C_max_ and AUC when the sublingual product was held in the mouth and not swallowed with saliva, suggesting that ketamine administered sublingually is substantially absorbed in the gastrointestinal tract after swallowing (Yanagihara et al. 2003). Also requiring urgent attention is the development of recommended treatment guidelines for oral administration, as this route is likely to be favoured by mental health clinicians utilising ketamine in an off-label capacity, due to its ease of use and favourable safety profile. These often-solitary practitioners must be able to look to the literature for guidance in this regard and the current lack of consensus as demonstrated by reported treatment regimens of multiple times a day to monthly, in oral ketamine studies is problematic, particularly given the current interest in off-label use in psychiatry. A dosing regimen which provides optimal benefits with least side effects should be considered through individual patient risk/benefit analysis (Swainson et al. 2022). Current evidence demonstrates a dose response effect in antidepressant efficacy, with repeated dosing shown to produce a robust and longer lasting effect, preclinically and clinically (Zarate and Machado-Vieira 2017), though this needs to be confirmed in larger prospective studies. Given a single dose is reported to induce a treatment effect for up to 2 weeks (Krystal et al. 2019), weekly dosing may offer efficacy, with a reduced risk of adverse effects. However, the effect of age and sex is yet to be fully elucidated in clinical populations, though preclinical models indicate oestrogen receptor activity results in enhanced sensitivity to ketamine’s antidepressant effects and this may be mediated by oestradiol facilitation of transcriptional inductive expression of AMPA receptors (Ho et al. 2018). Additionally, clinically, women are shown to experience higher rates of MDD and sex-specific patterns of glutamate receptor gene expression may contribute to this (Gray et al. 2015), whilst oestrogen mediated sensitivity may also contribute to treatment response and this area also requires future exploration.

In conclusion, emerging evidence suggests oral ketamine is a viable mental health treatment option; however, further research is required to provide a greater pharmacological understanding of oral ketamine, to inform dosing regimens for effective treatment and patient safety. Considering the current lack of evidence and consensus regarding a dosing protocol for oral ketamine, individually titrated dosing, within a risk versus benefit framework, is likely to be necessary to maximise both clinical response and safety and tolerability.
